# Association of *BMAL1 clock* gene polymorphisms with fasting glucose in children

**DOI:** 10.1038/s41390-023-02467-8

**Published:** 2023-02-02

**Authors:** Yi-De Yang, Yuan Zeng, Jian Li, Jun-Hua Zhou, Quan-Yuan He, Chan-Juan Zheng, Christoph Reichetzeder, Bernhard K. Krämer, Berthold Hocher

**Affiliations:** 1 Department of Child and Adolescent Health, School of Medicine, Hunan Normal University, 410006 Changsha, China; 2 Key Laboratory of Molecular Epidemiology of Hunan Province, School of Medicine, Hunan Normal University, 410081 Changsha, China; 3 Key Laboratory of Study and Discovery of Small Targeted Molecules of Hunan Province, School of Medicine, Hunan Normal University, 410013 Changsha, China; 4 Institute of Nutritional Science, University of Potsdam, Potsdam, Germany; 5 HMU – Health and Medical University, Potsdam, Germany; 6 Fifth Department of Medicine (Nephrology/Endocrinology/Rheumatology), University Medical Centre Mannheim, University of Heidelberg, Mannheim, Germany; 7 Reproductive and Genetic Hospital of CITIC-Xiangya, Changsha, China; 8 Institute of Medical Diagnostics, IMD Berlin, Berlin, Germany

## Abstract

**Background:**

The brain and muscle Arnt-like protein-1 (*BMAL1*) gene is an important circadian clock gene and previous studies have found that certain polymorphisms are associated with type 2 diabetes in adults. However, it remains unknown if such polymorphisms can affect fasting glucose in children and if other factors modify the associations.

**Methods:**

A school-based cross-sectional study with 947 Chinese children was conducted. A multivariable linear regression model was used to analyze the association between *BMAL1* gene polymorphisms and fasting glucose level.

**Results:**

After adjusting for age, sex, body mass index (BMI), physical activity, and unhealthy diet, GG genotype carriers of *BMAL1* rs3789327 had higher fasting glucose than AA/GA genotype carriers (*b* = 0.101, SE = 0.050, *P* = 0.045). Adjusting for the same confounders, rs3816358 was shown to be significantly associated with fasting glucose (*b* = 0.060, SE = 0.028, *P* = 0.032). Furthermore, a significant interaction between rs3789327 and nutritional status on fasting glucose was identified (*P*_interaction_ = 0.009); rs3789327 was associated with fasting glucose in the overweight/obese subgroup (*b* = 0.353, SE = 0.126, *P* = 0.006), but not in non-overweight/non-obese children.

**Conclusions:**

*BMAL1* polymorphisms were significantly associated with the fasting glucose level in children. Additionally, the observed interaction between nutritional status and *BMAL1* supports promoting an optimal BMI in children genetically predisposed to higher glucose level.

**Impact:**

- Polymorphisms in the essential circadian clock gene *BMAL1* were associated with fasting blood glucose levels in children. Additionally, there was a significant interaction between nutritional status and *BMAL1* affecting fasting glucose levels.
- *BMAL1* rs3789327 was associated with fasting glucose only in overweight/obese children.
- This finding could bring novel insights into mechanisms by which nutritional status influences fasting glucose in children.

## Introduction

It is estimated that the prevalence of diabetes in the global adult population in 2021 is approximately 10.5% (536.6 million), which is projected to rise to 12.2% (783.2 million) by 2045.^1^ A similar rising trend can be observed regarding the prevalence of impaired fasting glucose in children or adolescents.^2,3^ In China, the prevalence of impaired/abnormal fasting glucose and diabetes in children aged 6–17 was 1.89 and 0.1% in 2014, respectively.^4^ In addition, the earlier the onset of diabetes, the longer the patient’s lifetime exposure to hyperglycemia, the more destructive the disease is and the more likely it is going to lead to the earlier development of diabetes related complications reducing the overall quality of life.^5^ This indicates that it is necessary to explore the causes of hyperglycemia in children and adolescents, which will be beneficial to an early-life prevention and control of diabetes at childhood. A Chinese and Danish cross-population twin study demonstrated a high genetic influence on fasting glucose levels, with heritability ranging from 55 to 71%.^6^ However, blood glucose levels are influenced by both environmental and genetic factors, and the gene–environment interaction is also very important.^7,8^

Many aspects of physiology and behavior, such as the daily rhythms of food intake, metabolism, and, more specifically, glucose metabolism are regulated by the circadian clock system.^9^ Brain and muscle Arnt-like protein-1 (*BMAL1*) is an important component of the circadian clock in mammals and controls the oscillations of the circadian rhythm. It has been shown that disruptions of circadian rhythm oscillations in glucose metabolism are involved in the pathogenesis of type 2 diabetes.^10^ Sadacca et al. demonstrated that *BMAL1* is essential for normal insulin secretion and glucose homeostasis in pancreatic beta cells.^11^ Additionally, an experimental study suggested that obesity and diabetes may reduce the rhythmic expression of clock genes in the liver and adipose tissue.^12^ Population studies about *BMAL1* gene polymorphisms and risk of type 2 diabetes were controversial and all conducted in adults.^13–15^ Until now, the relation between *BMAL1* gene polymorphisms and fasting glucose levels in children is not clear. Therefore, the purpose of this study was to investigate the relationship between *BMAL1* gene polymorphisms and fasting glucose levels in Chinese children.

In addition, whether *BMAL1* polymorphisms could interact with nutritional status affecting fasting glucose has not been demonstrated before. Thus, we also investigated the interaction between *BMAL1* gene polymorphisms and nutritional status on fasting glucose levels in Chinese children.

## Subjects and methods

### Subjects

Using a cluster sampling method, a total of 1019 children aged 10–15 years from 3 middle schools in Changsha city, Hunan Province, China were included. The study was conducted in 2019. Seven participants were excluded for invalid questionnaire data. Sixty-five participants were excluded for absence of glucose phenotype data or genotype data. Therefore, a total of 947 participants were included in the present study. The study was approved by the Medical Ethic Committee of Hunan Normal University. Written informed consents was obtained from all participants and their parents.

### Measurement

Height and weight were measured according to standard protocols. Peripheral venous blood sample were collected under an overnight fasting condition. Then the fasting plasma blood glucose was measured using GOD-PAP method with the auto analyzer OLYMPUS AU400 with a standard protocol. For the fasting requirement, parents of the included children were informed one day before the blood collection by text message, and also during the blood collection, the nurses confirmed the fasting status of the participants. Covariates, including demographic characteristics and lifestyle factors, such as physical activity and dietary behaviors, were investigated by questionnaire.^16,17^ Dietary behaviors included fried chips/cakes/cookies and soft drinks. Participants were given the option of “eating/drinking in the past 7 days” (Yes), or “not eating/drinking in the past 7 days” (No). According to the “Dietary Guidelines for Chinese School-Age Children (2022)”, the physical activity was divided into “<1 h/day” and “≥1 h/day”.^16,18^

According to fasting blood glucose, the definition of prediabetes was 5.6–6.9 mmol/L and diabetes was ≥7.0 mmol/L in Chinese children.^19^ Also, the stratified association between *BMAL1* gene polymorphisms and blood glucose level were analyzed with multiple linear regression models in children with different glucose status (normal blood glucose and prediabetes/diabetes).

Body mass Index (BMI) was calculated by weight divided by square of height (kg/m^2^). Nutritional status of children was defined according to Working Group of Obesity in China (WGOC) criteria, of which the age and sex-specific cut-off points are the 85th and 95th percentiles of BMI (Overweight: BMI ≥ 85th but <90th percentile; Obese: BMI ≥ 90th percentile).^20^

### Genetic polymorphisms selection and genotyping

Selection of the BMAL1 gene polymorphisms was based on previous literature regarding population studies on the BMAL1 gene and cardiometabolic risk factors. Polymorphisms with significant association with cardiometabolic risk factors were selected. Then, we used the 1000 genome database to identify the MAF of the SNPs in East Asians and only SNPs with MAF > 0.05 were included in the present study. Finally, four SNPs from the *BMAL1* gene were selected (rs10832020,^21^ rs3789327,^13^ rs7950226,^14,15^ and rs3816358^22^).

Genomic DNA of peripheral blood leukocytes were extracted from participants’ fasting venous blood employing a salt extraction method.^16,23^ Matrix-assisted laser desorption/ionization time of flight mass spectrometry (MALDI-TOF MS, Agena) was used for the genotyping of *BMALI* polymorphisms.^16^ Genotyping was performed by investigators who were blind to the participants’ phenotypes. All the call rates of the genotyping were above 98% (Table S1). In addition, the genotyping was conducted with 1% randomly selected duplicated DNA samples and the consistent rate of genotyping results were 100%.

### Statistical analyses

Hardy–Weinberg equilibrium was checked with the Chi-square test. *F*-statistics (*F*_ST_) was calculated using the formula *F*_ST_ = (*P*_1_ − *P*_2_)^2^/[(*P*_1_ + *P*_2_) × (2 − (*P*_1_ + *P*_2_))].^24^ *P*_1_ is the effect allele frequency of a gene polymorphism in the 1000 Genomes Project database of European ancestry and *P*_2_ indicates the gene frequency of the effect allele in this study. *F*_ST_ reflects the ancestral differences in the same gene polymorphism between the two populations. *F*_ST_ between 0 and 0.05 indicates a small ancestral difference; A range of 0.05–0.15 indicates a moderate ancestral difference; 0.15–0.25 indicates a large ancestral differences; a *F*_ST_ > 0.25 indicates very large ancestral difference.^25^ Descriptive statistical analysis was used to analyze the general demographic characteristics, genotypes and allele frequencies of the participants. *T* tests were used for continuous variables and Chi-square test was used for categorical variables. The best genetic models were selected according to the Akaike Information Criterion (AIC).^26^ Multivariable linear regression analysis was conducted to analyze the association between gene polymorphism and fasting glucose, and the regression coefficients (*b*) and standard error (SE) were presented. Model 1 is the crude model; for Model 2, we added sex, age, and BMI as covariates; and Model 3 included sex, age, BMI, physical activity, and unhealthy diet (including soft drink and consumption of fried chips/cakes/cookies) as covariates. Considering 4 SNPs of *BMAL1* were selected in the present study, we adjusted multiple testing for Bonferroni correction (*P* < 0.05/4 = 0.0125). SPSS for Windows (version 22.0, SPSS Inc., Chicago, IL) was used for statistical analysis.

## Results

### General characteristics

The general characteristics of the participants are shown in Table 1. A total of 947 children were included in the present study. The average age of the participants was 11.69 years, including 476 girls and 471 boys, respectively. Boys had significantly higher fasting glucose level and a higher percentage of being active (physical activity ≥2 h/day) than girls (*P* < 0.05). The prevalence of overweight and obesity in boys (24.6%) was significantly higher than that in girls (15.9%) (*P* = 0.001). For age, BMI, glucose status, unhealthy diet (including soft drink, fried chips/cakes/cookies consumption), and genotype frequency of *BMAL1* polymorphisms, no significant sex differences were found (*P* > 0.05). The ancestral differences in *BMAL1* gene polymorphisms between European population in the 1000 Genomes Project database and our population are all small (all *F*_ST_ values <0.05, Table S1).

**Table 1:** General characteristics of the present study.

Variables	Total (n = 947)	Boys (n = 471)	Girls (n = 476)	*P*
*Age (years)*	11.69 ± 0.66	11.74 ± 0.65	11.64 ± 0.67	0.150
*Glucose (mmol/L)*	4.88 ± 0.42	4.92 ± 0.40	4.84 ± 0.44	**0.007**
*BMI (kg/m*^*2*^*)*	17.69 (16.05, 20.21)	17.84 (16.07, 20.52)	17.60 (16.03, 19.81)	0.369
*Nutritional status*				**0.001**
Non-overweight/obesity	742 (79.8%)	350 (75.4%)	392 (84.1%)	
Overweight/obesity	188 (20.2%)	114 (24.6%)	74 (15.9%)	
*Glucose status*				0.053
Normal blood glucose	912 (96.3%)	449 (95.3%)	463 (97.3%)	
5.6–6.9 mmol/L (prediabetes)	33 (3.5%)	22 (4.7%)	11 (2.3%)	
≥7.0 mmol/L (diabetes)	2 (0.2%)	0	2 (0.4%)	
*Physical activity*				**<0.001**
≥1 h/day	344 (38.9%)	205 (45.8%)	139 (31.8%)	
<1 h/day	541 (61.1%)	243 (54.2%)	298 (68.2%)	
*Soft drink*				0.160
Yes	580 (61.2%)	299 (63.5%)	281 (59.0%)	
No	367 (38.8%)	172 (36.5%)	195 (41.0%)	
*Fried chips/cakes/cookies*				0.350
Yes	193 (21.8%)	103 (23.0%)	90 (20.5%)	
No	694 (78.2%)	344 (77.0%)	350 (79.5%)	
*rs10832020*				0.672
Genotyping [*n* (%)]				
TT	473 (50.1%)	241 (51.3%)	232 (48.9%)	
TC	375 (39.7%)	180 (38.3%)	195 (41.1%)	
CC	96 (10.2%)	49 (10.4%)	47 (9.9%)	
*rs3789327*				0.159
Genotyping [*n* (%)]				
AA	438 (46.4%)	226 (48.1%)	212 (44.7%)	
AG	417 (44.2%)	208 (44.3%)	209 (44.1%)	
GG	89 (9.4%)	36 (7.7%)	53 (11.2%)	
*rs7950226*				0.102
Genotyping [*n* (%)]				
AA	333 (35.8%)	169 (36.7%)	164 (35.0%)	
GA	458 (49.3%)	213 (46.3%)	245 (52.2%)	
GG	138 (14.9%)	78 (17%)	60 (12.8%)	
*rs3816358*				0.150
Genotyping [*n* (%)]				
GG	658 (69.9%)	342 (72.8%)	316 (66.9%)	
GT	260 (27.6%)	117 (24.9%)	143 (30.3%)	
TT	24 (2.5%)	11 (2.3%)	13 (2.8%)	

*BMI* body mass index.

*P* values <0.05 are set in bold.

### Association between *BMAL1* gene polymorphisms and fasting glucose levels

According to AIC criterion, a dominant genetic model was the best model for rs10832020, a recessive genetic model for rs3789327, and an additive genetic model for rs7950226 and rs3816358 (Table S2). Table 2 shows the associations between *BMAL1* polymorphisms and fasting glucose. In the recessive genetic model, using sex, age, BMI, physical activity, and unhealthy diet as covariates, a significant association between rs3789327 and fasting glucose was found. For the rs3789327 polymorphism, GG genotype carriers had higher blood glucose levels than GA/AA genotype carriers (*b* = 0.101, SE = 0.050, *P* = 0.045). In the additive genetic model, we found that rs3816358 polymorphism A allele was significantly associated with fasting glucose level after adjustment for sex, age, BMI, physical activity, and unhealthy diet (*b* = 0.060, SE = 0.028, *P* = 0.032). No significant associations between rs10832020 or rs7950226 and fasting glucose levels were found (*P* > 0.05). However, none of the polymorphisms were significantly associated with glucose level after correction for multiple comparison (*P* < 0.05/4 = 0.0125). We also analyzed the association between *BMAL1* gene polymorphisms and blood glucose level in children with different glucose status (normal blood glucose and prediabetes/diabetes), and neither in normal blood glucose group nor in children with prediabetes/diabetes participants significant association between the four SNPs of *BMAL1* and blood glucose level was found (*P* > 0.05, Table S3).

**Table 2:** Association of *BMAL1* gene polymorphism with fasting glucose level.

Models	SNP	*b*	SE	*P*
Model 1	rs10832020	−0.041	0.027	0.130
	rs3789327	0.101	0.047	**0.031**
	rs7950226	0.006	0.020	0.780
	rs3816358	0.052	0.026	**0.046**
Model 2	rs10832020	−0.045	0.027	0.102
	rs3789327	0.100	0.047	**0.033**
	rs7950226	0.006	0.020	0.782
	rs3816358	0.059	0.026	**0.026**
Model 3	rs10832020	−0.039	0.029	0.175
	rs3789327	0.101	0.050	**0.045**
	rs7950226	0.002	0.021	0.932
	rs3816358	0.060	0.028	**0.032**

Model 1 is the crude model; for Model 2, we add sex, age and BMI as covariates; and Model 3 with sex, age, BMI, physical activity, soft drink and fried chips/cakes/cookies as covariates.

*BMI* body mass index.

*P* values <0.05 are set in bold.

### Interaction between *BMAL1* gene polymorphisms and nutritional status on fasting glucose levels

Associations between *BMAL1* gene polymorphisms and fasting glucose stratified according to nutritional status are illustrated in Table 3.

**Table 3:** Interaction between nutritional status and *BMAL1* gene polymorphism in fasting glucose level.

SNP	Category	Genotype	*N*	Mean	SD	*b*	SE	*P*	*P*_interaction_
rs10832020	Non-overweight/obesity	TT	372	4.92	0.39				0.237
		TC + CC	369	4.86	0.43	−0.053	0.031	0.091	
	Overweight/obesity	TT	90	4.82	0.38				
		TC + CC	96	4.84	0.52	0.015	0.072	0.834	
rs3789327	Non-overweight/obesity	AA + AG	674	4.88	0.41				**0.009**
		GG	65	4.92	0.38	0.041	0.055	0.456	
	Overweight/obesity	AA + AG	170	4.80	0.37				
		GG	18	5.13	0.88	0.353	0.126	**0.006**	
rs7950226	Non-overweight/obesity	AA/GA/GG	269/359/98	4.88/4.88/4.91	0.37/0.39/0.50	−0.001	0.023	0.965	0.407
	Overweight/obesity	AA/GA/GG	60/91/35	4.80/4.87/4.82	0.40/0.51/0.39	0.011	0.052	0.831	
rs3816358	Non-overweight/obesity	CC/CA/AA	514/204/20	4.88/4.90/5.09	039/0.45/0.37	0.048	0.03	0.114	0.592
	Overweight/obesity	CC/CA/AA	136/48/3	4.81/4.90/5.11	0.39/0.61/0.33	0.099	0.074	0.183	

*SD* standard deviation, *SE* standard error.

*P* and *P*_interaction_ was adjusted for age, gender, physical activity, soft drink, and fried chips/cakes/cookies; *P* values <0.05 are set in bold.

**Fig. 1:**
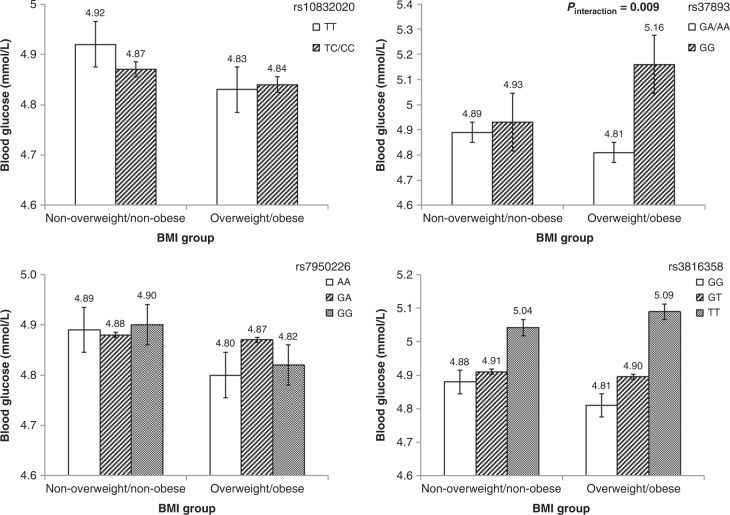
Adjusted means and standard errors of fasting glucose stratified by brain and muscle Arnt-like protein-1 (*BMAL1*) gene polymorphism phenotypes and nutritional status. BMI body mass index. Adjusted mean and standard errors were estimated under a general linear regression model that adjusted for age, sex, physical activity, and unhealthy diet.

A significant interaction between rs3789327 and nutritional status on fasting glucose was found (*P*_interaction_ = 0.009). In the overweight/obese subgroup, rs3789327 polymorphism GG genotype carriers had significantly higher fasting glucose than the AG/AA genotype carriers (*b* = 0.353, SE = 0.126, *P* = 0.006), but no significant association was examined in the subgroup who were not-overweight/non-obese. No significant interactions were observed for the rs10832020, rs7950226, and rs3816358 polymorphisms of *BMAL1*. The adjusted fasting glucose levels stratified by different genotypes and nutritional status are shown in Fig. 1.

## Discussion

To the best of our knowledge, this study is the first to demonstrate a significant association between the *BMAL1* rs3789327 and rs3816358 polymorphisms and fasting glucose levels in children. In addition, we found that there was a significant interaction between *BMAL1* rs3789327 polymorphism and nutritional status regarding fasting glucose levels. More specifically, the association between the rs3789327 polymorphism and fasting glucose levels only existed in overweight/obese children, not in children who were not overweight or obese. Our results suggest that *BMAL1* and nutritional status have an interacting effect on fasting blood glucose in children.

The finding of an association between the *BMAL1* rs3816358 polymorphism and fasting blood glucose in Chinese children is new. Although this SNP has not been reported in relation to fasting blood glucose or risk of diabetes, Leu et al. identified that the T allele of the rs3816358 polymorphism is associated with an increased risk of non-dipper hypertension (OR = 1.50, 95% CI: 1.04–2.18) in adults.^22^ In addition, Evans et al. found that the T allele of rs3816358 is significantly related with later sleep onset time (*b* = 0.13, SE = 0.05) objectively measured actigraphy in the Elderly in the United States.^27^ Previous studies reported that sleep onset time is significantly related with glucose-related phenotypes, such as diabetes, obesity, and fasting blood glucose, since later bedtime is likely to be associated with shorter sleep duration, leading to disruption of glycemic control or glycemic rhythmicity.^28,29^ But in the present study, the sleep traits of children were not investigated, which limited us to explore the potential mediation effect of sleep onset time in the association between rs3816358 and fasting blood glucose. Future studies could be conducted to further characterize this mediation effect.

In addition, our study shows that the GG genotype of rs3789327 might be a risk genotype for high fasting glucose levels in children. Previous studies have focused on different readouts, demonstrating associations with myocardial infarction, multiple sclerosis, and depression.^30–32^ This is the first study detecting association between rs3789327 polymorphisms and glycemia related phenotypes. However, the significant association was not significant after correcting for multiple testing. The findings should be interpreted with caution, and larger sample size studies and functional studies are needed to verify these results.

The current study did not detect significant associations between rs7950226/rs10832020 and fasting glucose. In addition, we also analyzed the association between *BMAL1* gene polymorphisms and blood glucose levels in normal blood glucose and prediabetes/diabetes participants, no significant results were found. Several previous studies have shown that *BMAL1* gene polymorphisms are associated with the risk of developing type 2 diabetes in adults.^13–15^ However, studies have noted that *BMAL1* gene polymorphisms are not associated with the occurrence of type 2 diabetes in Japanese^33^ and African-American^34^ cohorts. In contrast to the findings of our study, Pappa et al. showed that rs7950226 polymorphism of *BMAL1* gene is associated with susceptibility to gestational diabetes in Greek pregnant women.^14^ However, this might be due to the different age, ethnic diversity and varying environmental conditions of the study population. Furthermore, different genetic risk factors might exist in different population with different ethnicity.

Regarding potential mechanism underlying the associations between BMAL1 gene polymorphisms and fasting glucose, it is noteworthy that *BMAL1* acts an essential component of the circadian oscillation, which drives the daily rhythms of physiology and behavior.^35^ In pancreatic β cells, BMAL1 plays a key role in mediating insulin secretion, exocytosis and metabolism.^36^ According to previous functional studies about clock genes and glycemic phenotypes, the possible link between *BMAL1* and fasting glucose levels might be mitochondrial dysfunction.^37–39^Mitochondrial dysfunction is increasingly considered to be the main driver of pancreatic β cell failure in the pathogenesis of diabetes mellitus, and the loss of normal β cell function is the core factor of impaired insulin secretion in diabetes mellitus.^40^

Furthermore, results of the current study demonstrated a significant interacting effect between nutritional status and rs3789327 on fasting blood glucose levels. In overweight/obese individuals, GG genotype carriers had significantly higher levels of fasting blood glucose than AA/AG genotype carriers, which was not detected in children without overweight/obesity. To the best of our knowledge, no gene–nutritional status interaction of *BMAL1* gene was reported before. However, recent studies have found an association between *BMAL1* and indicators of obesity and obesity is closely associated with elevated fasting glucose in children.^41,42^ Being overweight or obese might amplify the genetic susceptibility of unfavorable glucose level for specific genotype carriers of BMAL1 gene polymorphism. Previous studies have reported that glucose-related phenotypes (Type 2 diabetes) which are attributed to genetic predisposition can be significantly different in people with different nutritional status.^43,44^ The identified interaction of rs3789327 with nutritional status on glucose in our study is of clinical interest, considering that mounting evidence have shown that fasting glucose levels in childhood are significant predictors for diabetes and other related cardiometabolic risk factors in adulthood.^45,46^ Mechanistically, studies have demonstrated that *BMAL1* can activate *CRY* gene expression in conjunction with the *CLOCK* gene when *BMAL1* levels are high.^47^ The degradation of *CRY1* induces gluconeogenesis and maintains blood glucose levels, but high fat intake accelerates the degradation of autophagy *CRY1* and contributes to the development of obesity-related hyperglycemia. Furthermore, a recent study observed that *BMAL1* overexpression can enhance circadian clock function and β cell function, thus enhancing GSIS and systemic glucose metabolism in the context of diet-induced obesity.^48^ In short, the above-mentioned findings could imply that *BMAL1* gene polymorphisms and nutritional status have an essential role in affecting fasting blood glucose levels in children.

One strength of our study is that it focused on children. Previous studies have generally set their focus on the association of cardiovascular metabolic risk factors in adults with *BMAL1* gene polymorphisms and results have been controversial in different ethnic populations. Genetic studies during childhood, a period when environmental risk factors (alcohol drinking, smoking, etc.) are relatively minimal, could increase the possibility to detect a genetic risk factor that might otherwise be masked, therefore contribute to elucidate the etiology of abnormal glucose and early onset diabetes. Fasting blood glucose levels in children are closely related to genetic factors and an early onset of diabetes also dictates the development and severity of chronic complications. Children are also the ideal population for genetic association study of blood glucose.

However, there are also some limitations in our study. We only selected 4 representative SNPs of the *BMAL1* gene but there are far more known SNPs in this gene. Secondly, studies in adults have observed controversial results regarding the association between *BMAL1* gene and risk of diabetes in different ethnic groups. Therefore, further studies on children of other ethnic groups are needed. Finally, we did not investigate information about puberty among the study participants, so we were unable to explore the potential effect of puberty on fasting blood glucose levels.

In conclusion, our study demonstrates that *BMAL1* rs3789327 and rs3816358 polymorphisms are significantly associated with fasting blood glucose levels in Chinese children. We also found that rs3789327 was associated with fasting blood glucose in overweight/obese children, but not in non-overweight/non-obese children. In addition, nutritional status and rs3789327 had an interacting effect on fasting blood glucose levels. Our finding highlights the importance of promoting healthy nutrition, especially in children with a genetic susceptibility to higher fasting glucose. Thus, these findings might also contribute to the development of early prevention strategies for elevated blood glucose in children.

## Supplementary Information

Supplementary Tables

## Data Availability

The datasets analyzed in our study are available from the corresponding author on reasonable request.
