# Supplementary material for: Association of BMAL1 clock gene polymorphisms with fasting glucose in children
Source: Pediatr Res. 2023 Feb 2;94(2):653–9. doi: 10.1038/s41390-023-02467-8 (PMC10382306; doi:10.1038/s41390-023-02467-8)
Supplement: Supplementary file 1 — Supplementary Tables [file 41390_2023_2467_MOESM1_ESM.docx]

| **Table S1 Genotype distribution of *BMAL1* gene polymorphism and Hardy-Weinberg test** | | | | | | | | | | | |
| --- | --- | --- | --- | --- | --- | --- | --- | --- | --- | --- | --- |
| Gene | SNP | Chromosome | Position ^a^ | effect/other | EAF ^b^ | EAF ^c^ | *F_ST_ ^d^* | Genotype | Distribution | HWE P-value | Call rate |
| BMAL1 | rs10832020 | 11 | 13299796 | C/T | 0.300 | 0.156 | 0.029 | CC/TC/TT | 96/375/473 | 0.093 | 99.68% |
|  | rs3789327 | 11 | 13363769 | G/A | 0.315 | 0.474 | 0.026 | GG/GA/AA | 89/417/438 | 0.473 | 99.68% |
|  | rs7950226 | 11 | 13296592 | G/A | 0.395 | 0.536 | 0.020 | GG/GA/AA | 138/458/333 | 0.338 | 98.10% |
|  | rs3816358 | 11 | 13369925 | A/C | 0.208 | 0.080 | 0.033 | AA/AC/CC | 24/260/458 | 0.076 | 99.47% |
| ^a^ Position: GRCh38 version. | | | | | | | | | | | |
| ^b^ Effect allele frequency in this study | | | | | | | | | | | |
| ^c^ Effect allele frequency in 1000 Genomes Project database of Europe populations | | | | | | | | | | | |
| ^d^ Effector allele frequencies of polymorphisms in this study population and ethnic differences in Europe populations | | | | | | | | | | | |

| **Table S2. Association of *BMAL1* gene polymorphism with blood glucose level** | | | | | | | |
| --- | --- | --- | --- | --- | --- | --- | --- |
| SNP | Genetic model | Model 1 | | | | | |
|  |  | B | SE | β | t | *P* | AIC |
| rs10832020 | Additive model | -0.029 | 0.020 | -0.047 | -1.435 | 0.152 | -1640.9974 |
|  | Dominant model | -0.041 | 0.027 | -0.049 | -1.515 | 0.130 | **-1641.2316** |
|  | Recessive model | -0.030 | 0.045 | -0.021 | -0.654 | 0.513 | -1639.3651 |
| rs3789327 | Additive model | 0.021 | 0.021 | 0.032 | 0.980 | 0.328 | -1640.7918 |
|  | Dominant model | 0.000 | 0.027 | 0.000 | 0.013 | 0.990 | -1639.8328 |
|  | Recessive model | 0.101 | 0.047 | 0.070 | 2.158 | **0.031** | **-1644.4882** |
| rs7950226 | Additive model | 0.006 | 0.020 | 0.009 | 0.279 | 0.780 | **-1631.5672** |
|  | Dominant model | 0.007 | 0.028 | 0.008 | 0.256 | 0.798 | -1631.5556 |
|  | Recessive model | 0.007 | 0.038 | 0.006 | 0.189 | 0.850 | -1631.5207 |
| rs3816358 | Additive model | 0.052 | 0.026 | 0.065 | 1.999 | **0.046** | **-1643.3501** |
|  | Dominant model | 0.042 | 0.030 | 0.046 | 1.407 | 0.160 | -1605.7187 |
|  | Recessive model | 0.216 | 0.086 | 0.081 | 2.506 | **0.012** | -1609.9534 |
| Mode l: not adjust any variables; SD standard deviation, B unstandardized, SE standard error. | | | | | | | |

| **Table S3. Association of *BMAL1* gene polymorphisms with blood glucose in children and adolescents stratified by glucose status** | | | | | | | | | | |
| --- | --- | --- | --- | --- | --- | --- | --- | --- | --- | --- |
| SNPs | Model | Normal blood glucose group | | | |  | Prediabetes/Diabetes group | | | |
|  |  | b | 95%CI | | *P* |  | b | 95%CI | | *P* |
| rs10832020 | Model 1 | -0.045 | -0.089 | 0.000 | 0.050 |  | 0.264 | -0.160 | 0.688 | 0.214 |
|  | Model 2 | -0.044 | -0.089 | 0.000 | 0.052 |  | 0.354 | -0.068 | 0.776 | 0.097 |
|  | Model 3 | -0.040 | -0.087 | 0.007 | 0.094 |  | 0.212 | -0.239 | 0.662 | 0.342 |
| rs3789327 | Model 1 | 0.053 | -0.024 | 0.131 | 0.178 |  | 0.272 | -0.290 | 0.834 | 0.332 |
|  | Model 2 | 0.059 | -0.018 | 0.137 | 0.133 |  | 0.213 | -0.419 | 0.845 | 0.497 |
|  | Model 3 | 0.050 | -0.033 | 0.133 | 0.234 |  | 0.038 | -0.641 | 0.717 | 0.909 |
| rs7950226 | Model 1 | -0.006 | -0.039 | 0.027 | 0.729 |  | 0.208 | -0.091 | 0.508 | 0.166 |
|  | Model 2 | -0.003 | -0.036 | 0.030 | 0.862 |  | 0.176 | -0.143 | 0.495 | 0.267 |
|  | Model 3 | -0.008 | -0.043 | 0.027 | 0.646 |  | 0.052 | -0.284 | 0.388 | 0.752 |
| rs3816358 | Model 1 | 0.029 | -0.014 | 0.072 | 0.189 |  | 0.247 | -0.112 | 0.605 | 0.171 |
|  | Model 2 | 0.033 | -0.011 | 0.076 | 0.140 |  | 0.235 | -0.120 | 0.590 | 0.186 |
|  | Model 3 | 0.034 | -0.012 | 0.080 | 0.151 |  | 0.163 | -0.234 | 0.561 | 0.405 |
| Model 1 is the crude model, for Model 2 we add sex, age and BMI as covariates, and Model 3 with sex, age, BMI, physical activity, soft drink and fried chips/cakes/cookies as covariates. BMI: body mass index. | | | | | | | | | | |
